# Genetic Defects in Phosphoinositide 3-Kinase δ Influence CD8^+^ T Cell Survival, Differentiation, and Function

**DOI:** 10.3389/fimmu.2018.01758

**Published:** 2018-08-02

**Authors:** Jennifer L. Cannons, Silvia Preite, Senta M. Kapnick, Gulbu Uzel, Pamela L. Schwartzberg

**Affiliations:** ^1^National Human Genome Research Institute, National Institutes of Health, Bethesda, MD, United States; ^2^National Institutes of Allergy and Infectious Disease, National Institutes of Health, Bethesda, MD, United States

**Keywords:** Epstein–Barr virus, activated phosphoinositide 3-kinase delta syndrome, p110δ activating mutation causing senescent T cells, lymphadenopathy and immunodeficiency, cytotoxic T lymphocyte, primary human immunodeficiency

## Abstract

Activated phosphoinositide 3-kinase delta syndrome (APDS), also known as p110 delta-activating mutation causing senescent T cells, lymphadenopathy and immunodeficiency (PASLI), is an autosomal dominant primary human immunodeficiency (PID) caused by heterozygous gain-of-function mutations in *PIK3CD*, which encodes the p110δ catalytic subunit of PI3K. This recently described PID is characterized by diverse and heterogeneous clinical manifestations that include recurrent respiratory infections, lymphoproliferation, progressive lymphopenia, and defective antibody responses. A major clinical manifestation observed in the NIH cohort of patients with *PIK3CD* mutations is chronic Epstein–Barr virus (EBV) and/or cytomegalovirus viremia. Despite uncontrolled EBV infection, many APDS/PASLI patients had normal or higher frequencies of EBV-specific CD8^+^ T cells. In this review, we discuss data pertaining to CD8^+^ T cell function in APDS/PASLI, including increased cell death, expression of exhaustion markers, and altered killing of autologous EBV-infected B cells, and how these and other data on PI3K provide insight into potential cellular defects that prevent clearance of chronic infections.

## Introduction

Cytotoxic CD8^+^ T lymphocytes (CTLs) are critical for the elimination of virally infected and tumor targets. Following T cell receptor (TCR) engagement in conjunction with cytokine signals, such as IL-2 and IL-12, naïve CD8^+^ T cells rapidly proliferate and differentiate from a “naïve” antigen-inexperienced state into an effector state characterized by the expression of cytolytic proteins (1). Upon subsequent engagement with targets, CTLs carry out their effector function through the directed release of cytoplasmic granules containing granzymes and other cytolytic effectors, as well as *via* cytokine secretion (1). CTLs tightly regulate the initiation and termination of granule secretion, a process critical for efficient and precise serial killing (2, 3).

After the resolution of infection, most CTLs are eliminated, although a fraction persist as long-lived memory cells to provide protection against subsequent pathogen encounter (4). However, in chronic infections where antigens persist over time, T cells can acquire an “exhausted” phenotype characterized by expression of inhibitory receptors that limit effector functions (5). While T cell exhaustion serves to dampen immune-mediated damage, it can also permit viral persistence and hinder anti-tumor responses (5). Recent data suggest that a small population of CD8^+^ T cells, marked by expression of the transcriptional regulator T cell factor 1 (TCF1), is required to maintain T cell responses during exhaustion in chronic infections (6–8).

The dynamic regulation of CD8^+^ T cell differentiation, proliferation, survival, and function is essential for generating effective immune responses. Mutations in genes affecting the function of CTLs and natural killer (NK) cells, an innate cell population that is also important for killing tumorigenic and virally infected cells, have been identified in numerous primary human immunodeficiencies (PIDs) associated with impaired viral clearance and tumor development (9). Such immunodeficiencies are also often associated with hemophagocytic syndrome, exemplified by secondary activation of the immune system in response to IFN-γ and other cytokines (9, 10). Thus, proper regulation of CTL function plays vital roles in both host protective immunity and immune cell homeostasis.

One condition where abnormal CD8^+^ T cell function can lead to substantial pathology is Epstein–Barr virus (EBV) infection. EBV is a common human gamma-herpesvirus that infects the oropharyngeal epithelium and B cells and is primarily controlled by CTLs and NK cell responses (11). Although infection in children is usually associated with mild symptoms, teenagers and adults can develop infectious mononucleosis with fever, enlarged secondary lymphoid organs, and flu-like symptoms, accompanied by a pronounced lymphocytosis, with increased CD8^+^ T cell numbers. In the normal host, Following initial infection, EBV persists latently in B cells. However, in immunocompromised patients, EBV can cause multiple severe complications that include lymphoproliferative disorders and lymphoid malignancies (12, 13).

Consistent with a critical role for CTLs in EBV control, as evidenced by the successful use of EBV-specific CTLs to treat EBV-induced disease after bone marrow transplantation (14), a growing number of PIDs have been associated with poor EBV clearance (10). Among these is the recently described autosomal-dominant immunodeficiency, activated phosphoinositide 3-kinase delta syndrome (APDS)/PASLI, associated with activating mutations affecting the p110δ catalytic subunit of phosphoinositide 3-kinase (PI3K) (15–19). PI3Ks are lipid kinases that are critical for the regulation of metabolism, differentiation, cell survival, and motility (20). Class Ia PI3Ks consist of two subunits, a regulatory subunit and a p110 catalytic subunit that phosphorylates phophosphoinositide PI(4,5)P_2_ to generate PI(3,4,5)P_3_, which recruits molecules to the plasma membrane, facilitating their activation. The p110δ catalytic isoform (encoded by *PIK3CD*) is expressed primarily in hematopoietic cells and is an important component of signaling pathways involved in T and B cell activation and differentiation in response to antigen, costimulatory, cytokine, and chemokine receptors (20).

**Figure 1:**
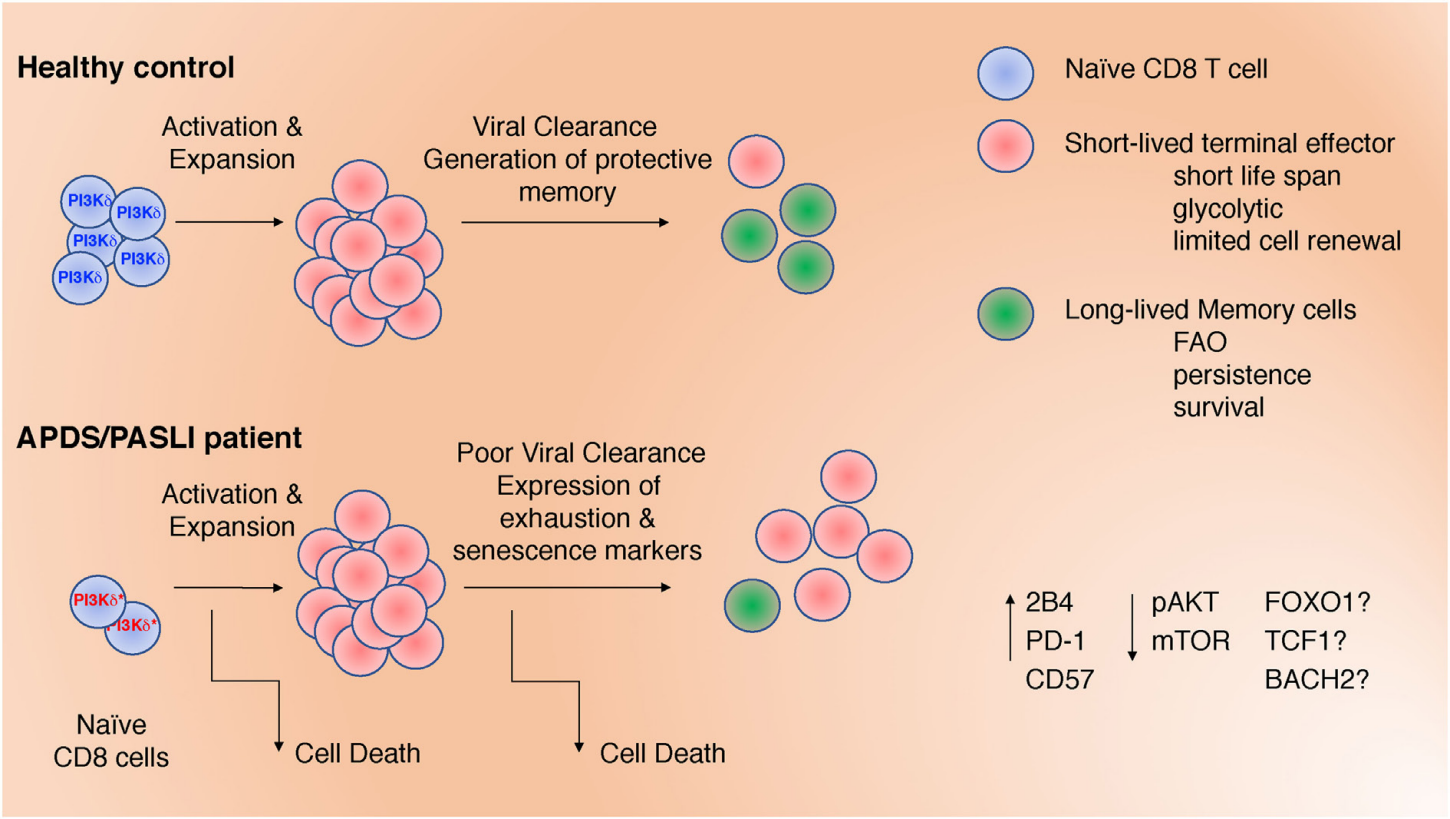
Defects in CD8^+^ T cells may contribute to impaired clearance of Epstein–Barr virus and CMV in activated phosphoinositide 3-kinase delta syndrome/PASLI. These include: (1) decreased naïve T cells; (2) increased T cell receptor-stimulated cell death; (3) altered differentiation with increased effector cell function at the expense of memory cell formation; and (4) expression of inhibitory receptors associated with exhaustion and/or senescence. These defects are associated with altered signaling (pAKT and mTOR) that, in turn, could affect the activation and/or expression of transcription factors (FOXO1, TCF1, and BACH2).

Activated phosphoinositide 3-kinase delta syndrome/PASLI is associated with frequent respiratory infections, progressive blood lymphopenia, mucosal lymphoid nodules, defective antibody responses, and lymphoma (15, 17–19, 21, 22). Patients have few naïve T cells, with evidence of increased T cell activation, whereas B cell development appears partially blocked, with few memory B cells (19, 23). Although recurrent respiratory infections are the most common feature of this PID (24), an inability to control viremia with EBV and cytomegalovirus (CMV) occurs in nearly half of all reported cases (16). Despite uncontrolled EBV viremia, many APDS/PASLI patients have normal or higher frequencies of EBV-specific CD8^+^ T cells, as detected by HLA tetramers loaded with lytic or latent EBV peptides (19). These data suggest that gain-of-function (GOF) mutations in p110δ do not result in a global impairment in the generation of antigen-specific T cell responses, raising the question of how activated p110δ affects CD8^+^ T cell differentiation, homeostasis, and function. Here, we discuss several features of CD8^+^ T cells in APDS/PASLI that may prevent clearance of EBV, including increased TCR-induced cell death, T cell exhaustion and immune senescence, and how activated PI3K might contribute to these phenotypes (Figure 1).

## CD8^+^ T Cell Death and Immune Homeostasis

Although APDS/PASLI patients can have increased percentages of EBV-specific CD8^+^ T cells (19), *in vitro* TCR stimulation results in pronounced cell death of both CD4^+^ and CD8^+^ T cells (15, 25). Thus, although abundant EBV-specific T cells are detected in the peripheral blood of APDS/PASLI patients, these cells may be more prone to death following re-stimulation. Instead of killing EBV-infected targets, CD8^+^ T cells may themselves die following TCR engagement and, therefore, not be able to clear the virus, particularly one that chronically remains in the body and continually “tickles” activated T cells.

How might PI3K/p110δ signaling affect TCR-mediated pro-apoptotic pathways? One of the main targets of PI3K activation is protein kinase B (AKT), which directly phosphorylates members of the Forkhead box O (FOXO) family of transcription factors resulting in their nuclear export and degradation (20, 26). Multiple FOXO transcriptional targets influence cell survival, both positively and negatively, depending on the cell type and experimental setting (26, 27). Although FOXO transcription factors drive the expression of genes encoding numerous cylin-dependent kinase inhibitors and the pro-apoptotic proteins BIM, PUMA, and FasL (26, 27), they can also suppress FasL expression in certain cell types (28). Deletion of *Foxo1* in murine T cells also decreases expression of *Il7ra*, which is important for T cell survival (29).

Additionally, the increased frequency of EBV positive cells in the peripheral blood may not accurately reflect tissue-specific frequency. PI3K regulates a number of molecules that affect lymphocyte recruitment and migration. Notably, FOXO1 transcriptional targets, such as *Ccr7* and *Kruppel-like factor 2* (*Klf2*) have profound effects on lymphocyte activation and trafficking in mice (29–31). CCR7 and its ligands play key roles in lymphocyte homing to the lymph nodes and intestinal Peyer’s patch (32). KLF2 is required for the effective transcription of *Sell* (encoding L-selection, CD62L) and *S1pr1* (encoding sphingosine-1-phosphate receptor-1, S1P_1_R), two key regulators of lymphocyte entry and egress from lymph nodes, respectively (33, 34). Notably, both CCR7 and CD62L are expressed at lower levels on T cells in peripheral blood from APDS/PASLI patients, which exhibit reduced CCR7^+^ naïve and central memory T cells, and a greater abundance of CD45RA^−^CCR7^−^ effector memory and CD45RA^+^CCR7^−^ terminal effector memory CD8^+^ T cells relative to controls (19).

A second major PI3K effector that influences lymphocyte migration and homeostasis is the mammalian target of Rapamycin, mTOR. MTOR is a conserved serine/threonine kinase that participates in two complexes, mTORC1 and 2. MTORC1 regulates cell growth, proliferation, survival, protein synthesis, and transcription (35, 36). Although some data convincingly argue that mTORC1 is not solely a PI3K effector in CTLs (37), T cells from APDS/PASLI patients show increased Rapamycin-sensitive phosphorylation of S6, a downstream target of the mTORC1 pathway (19). These data suggest that PI3K activation may be sufficient to activate mTORC1, even if it is not strictly required. Interestingly, mTORC1 and the downstream transcription factor hypoxia inducible factor 1-α (HIF1α) also affect expression of a large number of genes encoding chemokine and homing receptors. HIF1α-deficient murine T cells have higher expression of genes encoding CXCR4, CCR7, S1P_1_, and CD62L (37). The converse would be expected to occur in the presence of activated PI3Kδ. It is therefore of interest that APDS/PASLI patients are lymphopenic, yet have lymphadenopathy and splenomegaly as well as mucosal lymphoid nodules in their gastrointestinal and upper respiratory tracts, suggestive of altered lymphocyte homing (15, 19). Together, these data argue that continual PI3K signaling alters expression of key trafficking and survival proteins that influence the localization of T lymphocytes to tissues required for effective elimination of infection. Whether this affects responses to chronic infections, such as EBV, remains an interesting question.

## Altered CD8^+^ T Cell Differentiation

Despite the dramatic increase in TCR-induced cell death in APDS/PASLI patient CD8^+^ T cells, a fraction of blasts survived TCR stimulation *in vitro* and expand. Strikingly, these CD8^+^ T cell blasts displayed characteristics of enhanced effector function (19). Indeed, both AKT and mTOR are important for inducing and maintaining expression of cytolytic effector molecules in CTLs, including perforin and various granzymes (37, 38). These observations raise the question of whether continual PI3Kδ signaling promotes CD8^+^ T cell terminal effector differentiation.

Indeed, many transcription factors inhibited by PI3K activation, including FOXO1, TCF1, and BTB and CNC homology 2 (BACH2), influence CD8^+^ T cell differentiation. Mutations affecting these proteins promote effector T cell differentiation at the expense of memory formation (31, 39–43). Unequal PI3K signaling during cell division has been associated with bifurcation of sibling fates, with robust PI3K signaling promoting effector differentiation, associated with decreased expression of TCF1 (44). TCF1 is required for a CD8^+^ memory stem cell-like population that is necessary for continual responses to chronic infection (6–8), suggesting that reduced TCF1 due to sustained PI3K-signaling may prevent effective control of chronic viral infections.

**Figure 2:**
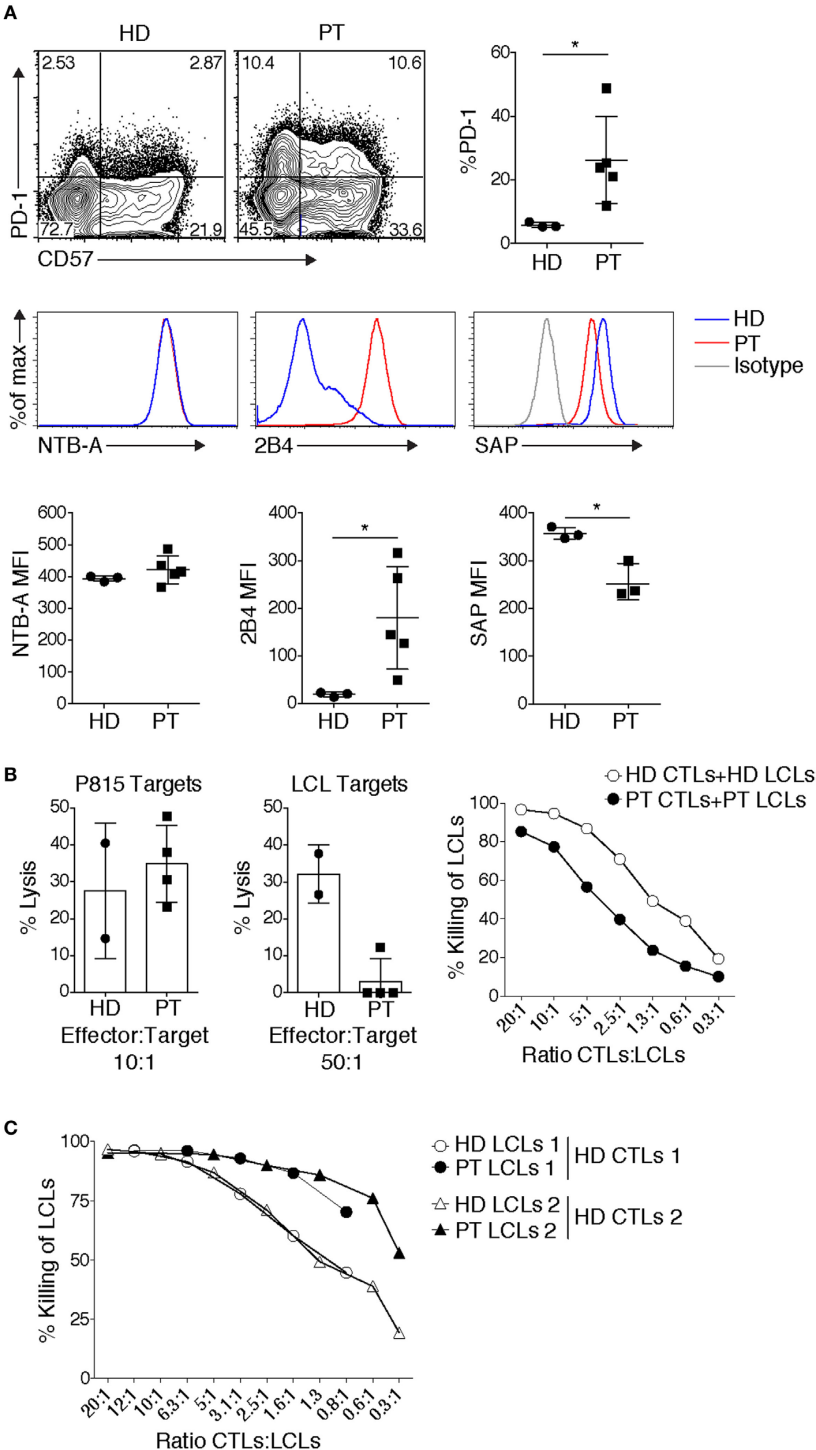
Patient CD8^+^ T cells display elevated expression of inhibitory receptors and impaired killing of autologous targets. **(A)** Elevated expression of inhibitory receptors PD-1 and 2B4, and senescence marker CD57 on allo-reactive CD8^+^ T cells. Expression of the signaling lymphocyte activation molecule family receptor, NTB-A, remained unchanged, while SAP expression can be reduced. Representative example shown [3 healthy donor (HD) controls, 3–5 patients] [PT], small horizon line represents mean, *p < 0.05 (Mann–Whitney test). **(B)** Defects in Epstein–Barr virus (EBV)-specific CD8^+^ T cells. Cytolysis of P815 targets by anti-CD3-mediated redirected lysis (left panel) and cytolysis of peptide-pulsed autologous LCLs (middle panel), (cytolysis from 2 HD controls and 4 patients cytotoxic T lymphocytes done in duplicate are shown, representative of 4 independent experiments). Right panel: an example of cytolysis of healthy donor (HD) and patient (PT) peptide-pulsed autologous LCLs, with titration of effector:target ratios. **(C)** HD EBV-specific CD8^+^ T cells cytolysis of HD or PT LCLs. Two examples are shown, which are representative of three independent experiments.

Other data have implicated HIF1α downstream of mTOR as critical for the expression of cytolytic effectors, including granzymes and perforin (37). Consistent with PI3K promoting mTORC1 activation, CD8^+^ T cell blasts from patients showed increased effector function, as determined by elevated IFN-γ production, and increased granzyme B expression and TCR-induced degranulation (19). Moreover, patient CTLs effectively killed the Fc receptor-expressing P815 murine target cell line coated with anti-CD3 in a re-directed lytic assay [Figure 2B and Ref. (19)]. Thus, APDS/PASLI patients have functional CTLs that even show evidence of enhanced effector cell function relative to controls.

How else might PI3K affect differentiation of CD8^+^ T cells? It is now appreciated that differentiation into effector cells is accompanied by major changes in cellular metabolism, associated with increased aerobic glycolysis (45). In contrast, memory cell formation is accompanied by increased use of fatty acid oxidation pathways, amino acid degradation, and a return to a more catabolic state (45). A number of metabolic and nutrient sensing pathways are mediated by PI3K and its downstream effectors: AKT and mTOR. AKT can induce the trafficking and surface expression of the glucose transporter, Glut1 (46, 47). Although the mechanism by which AKT alters Glut1 surface expression is still not clear, in other cell types this may occur *via* regulation of thioredoxin-interacting protein and inhibition of Glut1 internalization (48). Indeed, T cell blasts from APDS/PASLI patients demonstrate elevated glucose uptake compared to controls (19). Other data have implicated mTOR and HIF1α in the induction of genes encoding key glycolytic enzymes including hexokinase 2, phosphofructokinase, and pyruvate kinase, as well as Glut1 and Glut3 in CTLs (37). Whether differences in the metabolic profile of patient T cells contribute to, or are secondary to differences in their effector differentiation state and activation remains an open question. Nonetheless, these data point to a multi-faceted polarization to effector cells at the expense of long-term memory and efficient responses to chronic infection in the presence of activated PI3K.

## T Cell Exhaustion and/or Senescence

### T Cell Exhaustion

During chronic infections and/or persistent antigen exposure, T cell exhaustion can occur. Exhaustion manifests with several distinct features that include progressive loss of effector function, expression of multiple inhibitory receptors including PD-1, 2B4, Tim3, and LAG3, and an altered transcriptional program (5). In response to TCR stimulation, an elevated percentage of APDS/PASLI patient CD8^+^ T cells express PD-1 and 2B4 [Ref. (19, 25, 49) and Figure 2A], which may prevent effective CTL function.

2B4 is cytolytic receptor that is a member of the family of signaling lymphocyte activation molecule (SLAM) receptors, which associate with the small intracellular adaptor molecule SLAM-associated protein (SAP). Mutations affecting *SH2D1A*, which encodes SAP, cause X-linked lymphoproliferative disease type 1 (XLP1), which is perhaps the classic example of a PID associated with an inability to clear EBV (50–52). In the absence of SAP, or under conditions where SAP:2B4 ratios are low (53), 2B4 switches to function an inhibitory receptor, recruiting SH2-domain-containing tyrosine phosphatases 1 and 2 (SHP-1 and 2) and other negative signaling molecules, whose activities impair TCR signaling and subsequent T cell function (54–59). Because EBV-infected B cells express very high levels of CD48, the ligand for 2B4, killing of EBV-infected targets is specifically impaired in SAP-deficient (XLP1) NK and CTLs (50–52).

Although CD8^+^ EBV-specific T cells from APDS/PASLI patients killed P815 targets efficiently [Figure 2B and Ref. (19)], the P815 mouse mastocytoma cell line does not express ligands that stimulate human PD-1 and 2B4, preventing potential inhibitory effects of these receptors. In contrast, we and others found that patient CD8^+^ EBV-specific T cell blasts displayed variable defects in killing of autologous EBV-transformed lymphoblastoid B cell (LCL) targets [Figure 2B and Ref. (25)]. It is therefore of interest that in addition to high 2B4 levels, we have also observed reduced SAP levels in CTLs grown from APDS/PASLI patients (Figure 2A). We, therefore, propose that APDS/PASLI may share features with XLP1, with 2B4 acting as an inhibitory receptor that decreases killing EBV-infected B cells and possibly other hematopoietic cells infected by CMV. Notably, higher CD48 levels have been observed on APDS/PASLI patient B cells and LCLs compared to controls (25), which could also enhance 2B4 inhibitory signals. Interestingly, we have observed that control EBV-specific CTLs kill HLA-matched patient LCLs better than HLA-matched control LCLs, as might be expected if the increased CD48 on patient LCLs engage more 2B4, which acts as an activating receptor to enhance killing in control CTLs that express normal levels of SAP and 2B4. However, APDS/PASLI patients do not appear to develop the most severe phenotypes of XLP1, including hemophagocytic syndrome. This may be the result of less severe defects in cytolysis of EBV-infected B cells, other intrinsic T cell defects such as elevated cell death (15, 16) or alterations in macrophage activation (16) that may prevent secondary immune hyperactivation.

Intriguingly, 2B4 can also recruit SH2-containing inositol 5′ phosphastase (SHIP) (55), which hydrolyzes PI(3,4,5)P_3_ to PI(3,4)P_2_ (20). PD-1 can also dampen PI3K signals *via* the recruitment of phosphatases that preferentially downregulate signaling from CD28, a potent activator of PI3K (60). In addition, PD-1 ligation augments expression of PTEN (61), a lipid phosphastase that converts PIP_3_ to PI(4,5)P_2_, counteracting PI3K signaling (20). Thus, strong upregulation of PD-1 and 2B4 could serve as compensatory mechanisms to temper sustained PI3K activity, which may paradoxically result in greater defects in CD8^+^ T cell function.

### Senescence

In addition to expression of exhaustion markers, APDS/PASLI CD8^+^ T cells can also exhibit higher percentages expressing CD57, a marker of senescent T cells [Ref. (19, 25, 49) and Figure 2A]. During initial antigen encounter, T cell activation is followed by telomerase activation that preserves telomere length. However, subsequent TCR engagement can inactivate the telomerase promoter and decrease telomerase expression (62). Continual “tickling” of activated TCRs during chronic infections could result in telomere crisis and activation of DNA damage signals, followed by cell cycle arrest leading to replicative senescence or cell death (62). Although the elevated frequency of CD57^+^ T cells in APDS/PASLI could result from chronic infection, patients who were not overtly viremic also displayed an increased percentage of CD8^+^ T cells expressing CD57 (19). Alternatively, CD57 expression and detrimental telomere shortening observed in APDS/PASLI patient CD8^+^ T cells may reflect elevated basal PI3K signaling, evidenced by phosphorylated AKT and S6 (15, 19, 63). However, it has also been proposed that senescent cells survive for extended periods and are often more resistant to apoptotic cell death (62). This is not consistent with the observation that APDS/PASLI T cells rapidly die following *in vitro* activation (15). Nonetheless, these experiments collectively revealed that APDS/PASLI patients display CD8^+^ T cell dysfunction that includes features of both senescence and exhaustion that may contribute to their inability to clear chronic infections. Whether heterogeneity of these phenotypes is related to age of diagnosis, chronic infection, and/or environmental exposure remains an intriguing question as more patients are followed.

## Alternative Hypotheses

Although we have focused on CD8^+^ T cell function, alterations in other cells may also contribute to the inability to clear EBV, including recently documented defects in NK cell function (25, 64). Another hypothesis is that altered B cell development and populations may provide reservoirs for continual EBV infection in APDS/PASLI (65). It is important to highlight that Herpes viruses express proteins that converge on PI3K pathways to expidite viral entry, latency, and reactivation (66). Alternatively, although the role of humoral immunity in clearing EBV is not well defined, humoral defects may affect EBV infection in the context of immunodeficiency (67, 68). Altered properties of EBV-infected targets may contribute to poor EBV clearance. Sustained PI3K signals can rescue B cells from cell death in the absence of B cell receptor signaling (69); thus, increased PI3K signals may give EBV-infected B cells a survival advantage. However, we have found that LCLs from APDS/PASLI patients are actually killed better by control CTLs (Figure 2C). Finally, EBV occasionally infects T cells (70), and whether this affects CD8^+^ T cell function in APDS/PASLI remains unknown.

## Concluding Remarks

Here, we review some of the defects that may affect the ability of patients with APDS/PASLI to clear chronic infections such as EBV and CMV, with a focus on CD8^+^ T cells. A recent report has shown promising results using a PI3Kδ-specific inhibitor, Leniosilib, in a small group of APDS/PASLI patients (71). Inhibition of PI3Kδ rescued both T and B lymphocyte phenotypes, including decreased expression of activation, exhaustion and senescence T cell markers, and decreased lymphadenopathy and splenomegaly. Notably, Sirolimus treatment has also ameliorated lymphadenopathy and hepatosplenomegaly, and NK cell function in some APDS/PASLI patients, implicating mTOR in these phenotypes (19, 64, 72). How these treatments affect clearance of chronic infections is of great interest. However, recent evidence that treatment of mice with PI3Kδ inhibitors results in increased genomic instability in normal and neoplastic B cells (73) suggests that long-term PI3Kδ inhibitor administration could have detrimental consequences. Alternatives that boost T cell function may, therefore, be of continued interest for this disease. Further insight into cellular and molecular defects in APDS/PASLI is, therefore, an important component of understanding how to treat this complex disorder.

## Methods

### Samples and Ethics Approval

All human subjects in this study provided signed written informed consent in accordance with Helsinki principles for enrollment in research protocols that were approved by the Institutional Review Board of NIAID (clinical trials registration number NCT00001355, US NIH). Procedures were based on standard of care, under established clinical guidelines. Patients and control samples were described in Ref. (19). Peripheral blood mononuclear cells (PBMCs) were isolated using Ficoll–Hypaque gradient centrifugation.

### Flow Cytometry and Cytotoxicity

For phenotyping, cells were stained in FACS buffer (74). Staining reagents included: CD8-PECy7 (SK1), CD57-FITC (TB01), PD-1-PE (EBIOJ105), NTBA-PE (NT-7), 2B4-PE (C1.7), and SAP-PE (XLP1D12) (ebioscience). EBV-tetramers were kindly provided by Stuart Tangye. EBV peptides (HLA-A2-specific CLGGLLTMV and HLA-B8-specific RAKFKQLL) were from AnaSpec. Data were acquired on either a Calibur1 or LSRII flow cytometer (BD) and analyzed using FlowJo software (Tree Star). EBV-specific CTLs were generated from PBMCs that were pulsed with HLA-matched EBV-specific peptides (1 µg/ml) plus rhIL-2 (100 U/ml). Cytotoxicity assays were completed on day 10–12 of culture following assessment of outgrowth of antigen-specific cells *via* tetramer staining. *In vitro* cytolytic activity was determined using a lactate dehydrogenase release or flow-based assay as previously described in Ref. (74).

### Statistical Analysis

Data were analyzed *via* Prism 6 (GraphPad Software), using non-parametric unpaired Mann–Whitney *U* tests for comparison of two unpaired groups. *p* values <0.05 were considered statistically significant.

## Author Contributions

JC and SP performed experiments. GU provided patient samples and experimental advice. JC, SP, SK, and PS wrote the manuscript and helped prepared the figures. All authors edited and approved the manuscript.

## Conflict of Interest Statement

The authors declare that the research was conducted in the absence of any commercial or financial relationships that could be construed as a potential conflict of interest.

## References

[1] Stinchcombe JC Griffiths GM. Secretory mechanisms in cell-mediated cytotoxicity. Annu Rev Cell Dev Biol (2007) 23:495–517. 10.1146/annurev.cellbio.23.090506.123521 17506701

[2] Ritter AT Kapnick SM Murugesan S Schwartzberg PL Griffiths GM Lippincott-Schwartz J. Cortical actin recovery at the immunological synapse leads to termination of lytic granule secretion in cytotoxic T lymphocytes. Proc Natl Acad Sci U S A (2017) 114(32):E6585–94. 10.1073/pnas.1710751114 28716933 PMC5559056

[3] Stinchcombe JC Majorovits E Bossi G Fuller S Griffiths GM. Centrosome polarization delivers secretory granules to the immunological synapse. Nature (2006) 443(7110):462–5. 10.1038/nature05071 17006514

[4] Jameson SC Masopust D. Understanding subset diversity in T cell memory. Immunity (2018) 48(2):214–26. 10.1016/j.immuni.2018.02.010 29466754 PMC5863745

[5] Wherry EJ Kurachi M. Molecular and cellular insights into T cell exhaustion. Nat Rev Immunol (2015) 15(8):486–99. 10.1038/nri3862 26205583 PMC4889009

[6] Im SJ Hashimoto M Gerner MY Lee J Kissick HT Burger MC Defining CD8+ T cells that provide the proliferative burst after PD-1 therapy. Nature (2016) 537(7620):417–21. 10.1038/nature19330 27501248 PMC5297183

[7] Utzschneider DT Charmoy M Chennupati V Pousse L Ferreira DP Calderon-Copete S T cell factor 1-expressing memory-like CD8(+) T cells sustain the immune response to chronic viral infections. Immunity (2016) 45(2):415–27. 10.1016/j.immuni.2016.07.021 27533016

[8] Wu T Ji Y Moseman EA Xu HC Manglani M Kirby M The TCF1-Bcl6 axis counteracts type I interferon to repress exhaustion and maintain T cell stemness. Sci Immunol (2016) 1(6):eaai8593. 10.1126/sciimmunol.aai8593 28018990 PMC5179228

[9] Pachlopnik Schmid J Cote M Menager MM Burgess A Nehme N Menasche G Inherited defects in lymphocyte cytotoxic activity. Immunol Rev (2010) 235(1):10–23. 10.1111/j.0105-2896.2010.00890.x 20536552

[10] Cohen JI. Primary immunodeficiencies associated with EBV disease. Curr Top Microbiol Immunol (2015) 390(Pt 1):241–65. 10.1007/978-3-319-22822-8_10 26424649 PMC6349415

[11] Cohen JI Epstein-Barr virus infection. N Engl J Med (2000) 343(7):481–92. 10.1056/NEJM200008173430707 10944566

[12] Thorley-Lawson DA Gross A Persistence of the Epstein-Barr virus and the origins of associated lymphomas. N Engl J Med (2004) 350(13):1328–37. 10.1056/NEJMra032015 15044644

[13] Worth AJ Houldcroft CJ Booth C. Severe Epstein-Barr virus infection in primary immunodeficiency and the normal host. Br J Haematol (2016) 175(4):559–76. 10.1111/bjh.14339 27748521

[14] Liu Z Savoldo B Huls H Lopez T Gee A Wilson J Epstein-Barr virus (EBV)-specific cytotoxic T lymphocytes for the prevention and treatment of EBV-associated post-transplant lymphomas. Recent Results Cancer Res (2002) 159:123–33. 10.1007/978-3-642-56352-2_15 11785836

[15] Angulo I Vadas O Garcon F Banham-Hall E Plagnol V Leahy TR Phosphoinositide 3-kinase delta gene mutation predisposes to respiratory infection and airway damage. Science (2013) 342(6160):866–71. 10.1126/science.1243292 24136356 PMC3930011

[16] Carpier JM Lucas CL Epstein-Barr viurs susceptibility in activated PI3Kd syndrome (APDS) immunodeficiency. Front Immunol (2018) 8:2005 10.3389/fimmu.2017.02005 29387064 PMC5776011

[17] Coulter TI Chandra A Bacon CM Babar J Curtis J Screaton N Clinical spectrum and features of activated phosphoinositide 3-kinase delta syndrome: a large patient cohort study. J Allergy Clin Immunol (2017) 139(2):597–606.e4. 10.1016/j.jaci.2016.06.021 27555459 PMC5292996

[18] Jou ST Chien YH Yang YH Wang TC Shyur SD Chou CC Identification of variations in the human phosphoinositide 3-kinase p110delta gene in children with primary B-cell immunodeficiency of unknown aetiology. Int J Immunogenet (2006) 33(5):361–9. 10.1111/j.1744-313X.2006.00627.x 16984281

[19] Lucas CL Kuehn HS Zhao F Niemela JE Deenick EK Palendira U Dominant-activating germline mutations in the gene encoding the PI(3)K catalytic subunit p110delta result in T cell senescence and human immunodeficiency. Nat Immunol (2014) 15(1):88–97. 10.1038/ni.2771 24165795 PMC4209962

[20] Okkenhaug K. Signaling by the phosphoinositide 3-kinase family in immune cells. Annu Rev Immunol (2013) 31:675–704. 10.1146/annurev-immunol-032712-095946 23330955 PMC4516760

[21] Crank MC Grossman JK Moir S Pittaluga S Buckner CM Kardava L Mutations in PIK3CD can cause hyper IgM syndrome (HIGM) associated with increased cancer susceptibility. J Clin Immunol (2014) 34(3):272–6. 10.1007/s10875-014-0012-9 24610295 PMC4159085

[22] Kracker S Curtis J Ibrahim MA Sediva A Salisbury J Campr V Occurrence of B-cell lymphomas in patients with activated phosphoinositide 3-kinase delta syndrome. J Allergy Clin Immunol (2014) 134(1):233–6. 10.1016/j.jaci.2014.02.020 24698326 PMC4671279

[23] Dulau Florea AE Braylan RC Schafernak KT Williams KW Daub J Goyal RK Abnormal B-cell maturation in the bone marrow of patients with germline mutations in PIK3CD. J Allergy Clin Immunol (2017) 139(3):1032–5.e6. 10.1016/j.jaci.2016.08.028 27697496 PMC5342918

[24] Condliffe AM Chandra A Respiratory manifestations of the activated phosphoinositide 3-kinase delta syndrome. Front Immunol (2018) 9:338 10.3389/fimmu.2018.00338 29556229 PMC5844940

[25] Edwards ESJ Bier J Cole TS Wong M Hsu P Berflund LJ Activating PI3CD mutations impair cytotoxic lymphocyte differentiation, function and EBV immunity. J Allergy Clin Immunol (2018). 10.1016/j.jaci.2018.04.030 29800648

[26] Hedrick SM Hess Michelini R Doedens AL Goldrath AW Stone EL. FOXO transcription factors throughout T cell biology. Nat Rev Immunol (2012) 12(9):649–61. 10.1038/nri3278 22918467 PMC3875397

[27] Brunet A Bonni A Zigmond MJ Lin MZ Juo P Hu LS Akt promotes cell survival by phosphorylating and inhibiting a Forkhead transcription factor. Cell (1999) 96(6):857–68. 10.1016/S0092-8674(00)80595-4 10102273

[28] Jonsson H Allen P Peng SL. Inflammatory arthritis requires Foxo3a to prevent Fas ligand-induced neutrophil apoptosis. Nat Med (2005) 11(6):666–71. 10.1038/nm1248 15895074

[29] Kerdiles YM Beisner DR Tinoco R Dejean AS Castrillon DH DePinho RA Foxo1 links homing and survival of naive T cells by regulating L-selectin, CCR7 and interleukin 7 receptor. Nat Immunol (2009) 10(2):176–84. 10.1038/ni.1689 19136962 PMC2856471

[30] Carlson CM Endrizzi BT Wu J Ding X Weinreich MA Walsh ER Kruppel-like factor 2 regulates thymocyte and T-cell migration. Nature (2006) 442(7100):299–302. 10.1038/nature04882 16855590

[31] Kim MV Ouyang W Liao W Zhang MQ Li MO. The transcription factor Foxo1 controls central-memory CD8+ T cell responses to infection. Immunity (2013) 39(2):286–97. 10.1016/j.immuni.2013.07.013 23932570 PMC3809840

[32] Forster R Davalos-Misslitz AC Rot A. CCR7 and its ligands: balancing immunity and tolerance. Nat Rev Immunol (2008) 8(5):362–71. 10.1038/nri2297 18379575

[33] Arbones ML Ord DC Ley K Ratech H Maynard-Curry C Otten G Lymphocyte homing and leukocyte rolling and migration are impaired in L-selectin-deficient mice. Immunity (1994) 1(4):247–60. 10.1016/1074-7613(94)90076-0 7534203

[34] Matloubian M Lo CG Cinamon G Lesneski MJ Xu Y Brinkmann V Lymphocyte egress from thymus and peripheral lymphoid organs is dependent on S1P receptor 1. Nature (2004) 427(6972):355–60. 10.1038/nature02284 14737169

[35] Finlay D Cantrell DA. Metabolism, migration and memory in cytotoxic T cells. Nat Rev Immunol (2011) 11(2):109–17. 10.1038/nri2888 21233853 PMC3521506

[36] Saxton RA Sabatini DM mTOR signaling in growth, metabolism, and disease. Cell (2017) 169(2):361–71. 10.1016/j.cell.2017.03.035 28388417

[37] Finlay DK Rosenzweig E Sinclair LV Feijoo-Carnero C Hukelmann JL Rolf J PDK1 regulation of mTOR and hypoxia-inducible factor 1 integrate metabolism and migration of CD8+ T cells. J Exp Med (2012) 209(13):2441–53. 10.1084/jem.20112607 23183047 PMC3526360

[38] Macintyre AN Finlay D Preston G Sinclair LV Waugh CM Tamas P Protein kinase B controls transcriptional programs that direct cytotoxic T cell fate but is dispensable for T cell metabolism. Immunity (2011) 34(2):224–36. 10.1016/j.immuni.2011.01.012 21295499 PMC3052433

[39] Gattinoni L Zhong XS Palmer DC Ji Y Hinrichs CS Yu Z Wnt signaling arrests effector T cell differentiation and generates CD8+ memory stem cells. Nat Med (2009) 15(7):808–13. 10.1038/nm.1982 19525962 PMC2707501

[40] Hess Michelini R Doedens AL Goldrath AW Hedrick SM. Differentiation of CD8 memory T cells depends on Foxo1. J Exp Med (2013) 210(6):1189–200. 10.1084/jem.20130392 23712431 PMC3674697

[41] Jeannet G Boudousquie C Gardiol N Kang J Huelsken J Held W. Essential role of the Wnt pathway effector Tcf-1 for the establishment of functional CD8 T cell memory. Proc Natl Acad Sci U S A (2010) 107(21):9777–82. 10.1073/pnas.0914127107 20457902 PMC2906901

[42] Roychoudhuri R Clever D Li P Wakabayashi Y Quinn KM Klebanoff CA BACH2 regulates CD8(+) T cell differentiation by controlling access of AP-1 factors to enhancers. Nat Immunol (2016) 17(7):851–60. 10.1038/ni.3441 27158840 PMC4918801

[43] Zhou X Yu S Zhao DM Harty JT Badovinac VP Xue HH. Differentiation and persistence of memory CD8(+) T cells depend on T cell factor 1. Immunity (2010) 33(2):229–40. 10.1016/j.immuni.2010.08.002 20727791 PMC2928475

[44] Lin WH Adams WC Nish SA Chen YH Yen B Rothman NJ Asymmetric PI3K signaling driving developmental and regenerative cell fate bifurcation. Cell Rep (2015) 13(10):2203–18. 10.1016/j.celrep.2015.10.072 26628372 PMC4685001

[45] Buck MD Sowell RT Kaech SM Pearce EL Metabolic instruction of immunity. Cell (2017) 169(4):570–86. 10.1016/j.cell.2017.04.004 28475890 PMC5648021

[46] Jacobs SR Herman CE Maciver NJ Wofford JA Wieman HL Hammen JJ Glucose uptake is limiting in T cell activation and requires CD28-mediated Akt-dependent and independent pathways. J Immunol (2008) 180(7):4476–86. 10.4049/jimmunol.180.7.4476 18354169 PMC2593791

[47] Wieman HL Wofford JA Rathmell JC. Cytokine stimulation promotes glucose uptake via phosphatidylinositol-3 kinase/Akt regulation of Glut1 activity and trafficking. Mol Biol Cell (2007) 18(4):1437–46. 10.1091/mbc.e06-07-0593 17301289 PMC1838986

[48] Waldhart AN Dykstra H Peck AS Boguslawski EA Madaj ZB Wen J Phosphorylation of TXNIP by AKT mediates acute influx of glucose in response to insulin. Cell Rep (2017) 19(10):2005–13. 10.1016/j.celrep.2017.05.041 28591573 PMC5603216

[49] Wentink MWJ Mueller YM Dalm V Driessen GJ van Hagen PM van Montfrans JM Exhaustion of the CD8(+) T cell compartment in patients with mutations in phosphoinositide 3-kinase delta. Front Immunol (2018) 9:446 10.3389/fimmu.2018.00446 29563914 PMC5845988

[50] Cannons JL Tangye SG Schwartzberg PL. SLAM family receptors and SAP adaptors in immunity. Annu Rev Immunol (2011) 29:665–705. 10.1146/annurev-immunol-030409-101302 21219180

[51] Pachal N Booth C Cannons JL Schwartzberg PL X-linked lymphoproliferative disease type 1: a clinical and molecular perspective. Front Immunol (2018) 9:666 10.3389/fimmu.2018.00666 29670631 PMC5893764

[52] Tangye SG. XLP: clinical features and molecular etiology due to mutations in SH2D1A encoding SAP. J Clin Immunol (2014) 34(7):772–9. 10.1007/s10875-014-0083-7 25085526

[53] Chlewicki LK Velikovsky CA Balakrishnan V Mariuzza RA Kumar V. Molecular basis of the dual functions of 2B4 (CD244). J Immunol (2008) 180(12):8159–67. 10.4049/jimmunol.180.12.8159 18523281

[54] Bottino C Falco M Parolini S Marcenaro E Augugliaro R Sivori S NTB-A [correction of GNTB-A], a novel SH2D1A-associated surface molecule contributing to the inability of natural killer cells to kill Epstein-Barr virus-infected B cells in X-linked lymphoproliferative disease. J Exp Med (2001) 194(3):235–46. 10.1084/jem.194.3.235 11489943 PMC2193462

[55] Dong Z Davidson D Perez-Quintero LA Kurosaki T Swat W Veillette A. The adaptor SAP controls NK cell activation by regulating the enzymes Vav-1 and SHIP-1 and by enhancing conjugates with target cells. Immunity (2012) 36(6):974–85. 10.1016/j.immuni.2012.03.023 22683124

[56] Eissmann P Beauchamp L Wooters J Tilton JC Long EO Watzl C. Molecular basis for positive and negative signaling by the natural killer cell receptor 2B4 (CD244). Blood (2005) 105(12):4722–9. 10.1182/blood-2004-09-3796 15713798

[57] Parolini S Bottino C Falco M Augugliaro R Giliani S Franceschini R X-linked lymphoproliferative disease. 2B4 molecules displaying inhibitory rather than activating function are responsible for the inability of natural killer cells to kill Epstein-Barr virus-infected cells. J Exp Med (2000) 192(3):337–46. 10.1084/jem.192.3.337 10934222 PMC2193227

[58] Tangye SG Lazetic S Woollatt E Sutherland GR Lanier LL Phillips JH. Cutting edge: human 2B4, an activating NK cell receptor, recruits the protein tyrosine phosphatase SHP-2 and the adaptor signaling protein SAP. J Immunol (1999) 162(12):6981–5. 10358138

[59] Zhao F Cannons JL Dutta M Griffiths GM Schwartzberg PL. Positive and negative signaling through SLAM receptors regulate synapse organization and thresholds of cytolysis. Immunity (2012) 36(6):1003–16. 10.1016/j.immuni.2012.05.017 22683123 PMC3389133

[60] Hui E Cheung J Zhu J Su X Taylor MJ Wallweber HA T cell costimulatory receptor CD28 is a primary target for PD-1-mediated inhibition. Science (2017) 355(6332):1428–33. 10.1126/science.aaf1292 28280247 PMC6286077

[61] Wartewig T Kurgyis Z Keppler S Pechloff K Hameister E Ollinger R PD-1 is a haploinsufficient suppressor of T cell lymphomagenesis. Nature (2017) 552(7683):121–5. 10.1038/nature24649 29143824 PMC5821214

[62] Bellon M Nicot C. Telomere dynamics in immune senescence and exhaustion triggered by chronic viral infection. Viruses (2017) 9(10):E289. 10.3390/v9100289 28981470 PMC5691640

[63] Lucas CL Zhang Y Venida A Wang Y Hughes J McElwee J Heterozygous splice mutation in PIK3R1 causes human immunodeficiency with lymphoproliferation due to dominant activation of PI3K. J Exp Med (2014) 211(13):2537–47. 10.1084/jem.20141759 25488983 PMC4267241

[64] Ruiz-Garcia R Vargas-Hernandez A Chinn IK Angelo LS Cao TN Coban-Akdemir Z Mutations in PI3K110delta cause impaired natural killer cell function partially rescued by rapamycin treatment. J Allergy Clin Immunol (2018). 10.1016/j.jaci.2017.11.042 PMC6109967 29330011

[65] Wentink M Dalm V Lankester AC van Schouwenburg PA Scholvinck L Kalina T Genetic defects in PI3Kdelta affect B-cell differentiation and maturation leading to hypogammaglobulineamia and recurrent infections. Clin Immunol (2017) 176:77–86. 10.1016/j.clim.2017.01.004 28104464

[66] Cohen JI. Herpesviruses in the activated phosphatidylinositol-3-kinase-d syndrome. Front Immunol (2018) 9:237. 10.3389/fimmu.2018.00237 29599765 PMC5863522

[67] Bu W Hayes GM Liu H Gemmell L Schmeling DO Radecki P Kinetics of Epstein-Barr Virus (EBV) neutralizing and virus-specific antibodies after primary infection with EBV. Clin Vaccine Immunol (2016) 23(4):363–9. 10.1128/CVI.00674-15 26888186 PMC4820504

[68] Panikkar A Smith C Hislop A Tellam N Dasari V Hogquist KA Impaired Epstein-Barr virus-specific neutralizing antibody response during acute infectious mononucleosis is coincident with global B-cell dysfunction. J Virol (2015) 89(17):9137–41. 10.1128/JVI.01293-15 26109734 PMC4524077

[69] Werner M Hobeika E Jumaa H. Role of PI3K in the generation and survival of B cells. Immunol Rev (2010) 237(1):55–71. 10.1111/j.1600-065X.2010.00934.x 20727029

[70] Coleman CB Wohlford EM Smith NA King CA Ritchie JA Baresel PC Epstein-Barr virus type 2 latently infects T cells, inducing an atypical activation characterized by expression of lymphotactic cytokines. J Virol (2015) 89(4):2301–12. 10.1128/JVI.03001-14 25505080 PMC4338898

[71] Rao VK Webster S Dalm V Sediva A van Hagen PM Holland S Effective “activated PI3Kdelta syndrome”-targeted therapy with the PI3Kdelta inhibitor leniolisib. Blood (2017) 130(21):2307–16. 10.1182/blood-2017-08-801191 28972011 PMC5701526

[72] Rae W Ramakrishnan KA Gao Y Ashton-Key M Pengelly RJ Patel SV Precision treatment with sirolimus in a case of activated phosphoinositide 3-kinase delta syndrome. Clin Immunol (2016) 171:38–40. 10.1016/j.clim.2016.07.017 27444043

[73] Compagno M Wang Q Pighi C Cheong TC Meng FL Poggio T Phosphatidylinositol 3-kinase delta blockade increases genomic instability in B cells. Nature (2017) 542(7642):489–93. 10.1038/nature21406 28199309 PMC5382874

[74] Kapnick SM Stinchcombe JC Griffiths GM Schwartzberg PL Inducible T cell kinase regulates the acquisition of cytolytic capacity and degranulation in CD8(+) CTLs. J Immunol (2017) 198(7):2699–711. 10.4049/jimmunol.1601202 28213500 PMC5360469

